# Akt Inhibition Promotes Autophagy and Clearance of Group B *Streptococcus* from the Alveolar Epithelium

**DOI:** 10.3390/pathogens11101134

**Published:** 2022-09-30

**Authors:** Ioanna Pantazi, Iosif Papafragkos, Ourania Kolliniati, Ioanna Lapi, Christos Tsatsanis, Eleni Vergadi

**Affiliations:** 1 Laboratory of Clinical Chemistry, School of Medicine, University of Crete, 71003 Heraklion, Greece; 2 Institute of Molecular Biology and Biotechnology, FORTH, 71100 Heraklion, Greece; 3 Department of Mother and Child, School of Medicine, University of Crete, 71003 Heraklion, Greece

**Keywords:** Group B *Streptococcus*, epithelial cells, lung, alveolar, Akt, mTOR, autophagy, neonate

## Abstract

Group B *Streptococcus* (GBS) is a gram-positive bacterium that is harmless for healthy individuals but may provoke invasive disease in young infants and immunocompromised hosts. GBS invades the epithelial barriers to enter the bloodstream, and thus strategies that enhance epithelial cell responses may hamper GBS invasion. In the present study, we sought to investigate whether the inhibition of Akt, a kinase that regulates host inflammatory responses and autophagy via suppression of mTOR, can enhance the response of non-phagocytic alveolar epithelial cells against GBS. Treatment of the alveolar epithelial cell line A549 with the Akt inhibitor MK-2206 resulted in the enhanced production of reactive oxygen species and inflammatory mediators in response to GBS. Additionally, Akt inhibition via MK-2206 resulted in elevated LC3II/I ratios and increased autophagic flux in alveolar epithelial cells. Importantly, the inhibition of Akt promoted GBS clearance both in alveolar epithelial cells *in vitro* and in lung tissue in vivo in a murine model of GBS pneumonia. The induction of autophagy was essential for GBS clearance in MK-2206 treated cells, as knockdown of ATG5, a critical component of autophagy, abrogated the effect of Akt inhibition on GBS clearance. Our findings highlight the role of Akt kinase inhibition in promoting autophagy and GBS clearance in the alveolar epithelium. The inhibition of Akt may serve as a promising measure to strengthen epithelial barriers and prevent GBS invasion in susceptible hosts.

## 1. Introduction

Group B *Streptococcus* (GBS or *Streptococcus agalactiae*) is a gram-positive encapsulated bacterium that is usually a commensal for healthy individuals and part of the normal flora of the intestine or the lower genital tract of approximately 6–36% of women [1,2]. However, GBS may provoke severe invasive disease in vulnerable groups such as the elderly, pregnant women, and immunocompromised hosts, especially newborns and young infants, causing pneumonia, sepsis and meningitis [1,3]. 

Newborns are usually infected by GBS during vaginal delivery; either via the aspiration of infected amniotic or vaginal fluids, or via the colonization of their nasopharyngeal epithelium during direct contact with the GBS-colonised vaginal canal [1,4,5]. The alveolar epithelium provides the first line of defense against invading pathogens, as it constitutes a physical barrier, produces antibacterial compounds, is capable of non-professional phagocytosis, bacteria killing, as well as the recruitment and activation of immune cells [6]. However, in susceptible hosts, GBS is capable of invading epithelial and endothelial cells and entering the bloodstream [7,8]. Additionally, GBS can survive inside respiratory epithelial cells, and, via this method, may gain access into the blood circulation [4,5,6,7,8]. Today, the defense mechanisms that are utilized by the lung epithelium against GBS are unknown. 

Autophagy is an evolutionarily conserved homeostatic process that utilizes the lysosomal machinery for the removal and recycling of damaged cytosolic components [9,10]. Antimicrobial autophagy, also known as xenophagy, is a type of selective autophagy that utilizes the components of the autophagic machinery to eliminate intracellular bacteria [9,11,12,13,14]. Specifically, in antimicrobial autophagy, elements of the canonical autophagy conjugate microtubule-associated protein light chain 3 (LC3II) to the membranes of phagosomes, facilitating rapid phagosome maturation and lysosomal fusion [8,15,16]. Autophagy is known to be negatively regulated by the PI3K/Akt/mammalian target of the rapamycin (mTOR) pathway. Specifically, activated Akt kinase inhibits the activity of tuberous sclerosis complex 1/2 (TSC1/2), allowing the Ras homolog enriched in brain (Rheb) to activate mTOR complex 1 (mTORC1), which then promotes the inhibition of autophagy [17,18]. Earlier studies from our group demonstrated that the inhibition of Akt1 results in the enhanced activation of macrophages in response to toll-like receptor (TLR)-4 signals, [19] and recent studies have shown that the inhibition of Akt kinase plays a crucial role in the promotion of bacterial clearance in macrophages via reducing mTORC1 and Rheb and promoting autophagy [20,21,22].

Today, little is known about the host defenses employed by the lung epithelium against GBS and whether we can enhance the bactericidal capacity of lung epithelial cells against GBS. Non-phagocytic cells have the potential to eliminate intracellular bacteria that escape from the endocytic pathway to the cytoplasm via activation of autophagic pathways [12,23]. However, it remains unknown whether this molecular mechanism can be utilized by non-professional phagocytes, such as alveolar epithelial cells during GBS infection, to assist in bacteria clearance and hamper blood invasion and dissemination. In this study, we investigated whether Akt inhibition promotes autophagy and enhances epithelial cell host defense as a potential approach to facilitate GBS elimination and preventing invasive disease. 

## 2. Results

### 2.1. GBS Infection Activates Akt in Alveolar Epithelial Cells

**Figure 1:**
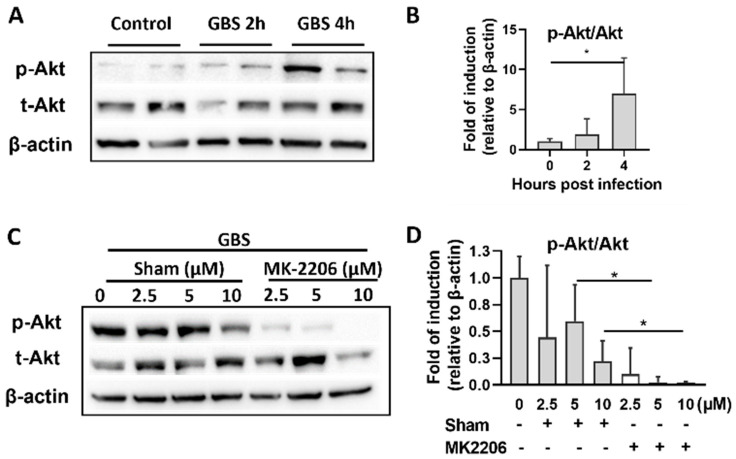
**Activation of Akt upon Group B *Streptococcus* (GBS) infection.** (**A**,**B**). Alveolar epithelial (A549) cells were infected with GBS and both control and GBS infected cells were collected two and four hours post infection. Representative western blot analysis and densitometry analysis of all replicates of phosphorylated (p-Akt1) and total Akt (t-Akt) protein levels are depicted. (**C**,**D**). Alveolar epithelial A549 cells were treated with different concentrations of the specific Akt inhibitor MK-2206 or its diluent (sham) (2.5, 5, 10 μΜ) for 24 h and GBS infection followed for 2 h. Representative western blot analysis and densitometry analysis of all replicates of p-Akt1 and total Akt protein levels are depicted. Densitometry analyses are plotted in bar graphs as median +/- interquartile range. A statistical analysis was performed with Kruskal-Wallis (**B**) and Mann-Whitney test ((**D**), between MK-2206 and Sham treated cells at each time point for 5 μΜ or 10 μΜ concentrations). n = 4–5 per group, * *p* < 0.05.

The Akt pathway is activated by several extracellular stimuli such as cytokines, growth factors and pathogens [24]. To investigate whether Akt signaling is activated by GBS, we exposed A549 cells to GBS for 2 h and then removed extracellular GBS following treatment with antibiotic containing media. Cell lysates were collected at 2 and 4 h post infection. Activation of Akt was assessed by measuring its phosphorylation level. Western blot analysis showed a significant increase in Akt phosphorylation 2 and 4 h following GBS infection (Figure 1A,B). Treatment with Akt inhibitor MK-2206 (at 5 or 10 μΜ) resulted in inhibition of Akt phosphorylation (Figure 1C,D). Concentration of 5μΜ was selected for further cell culture experiments since it provoked effective inhibition without affecting cell proliferation (Supplementary Figure S1).

### 2.2. Inhibition of Akt Kinase Promotes GBS Clearance by Lung Epithelial Cells

**Figure 2:**
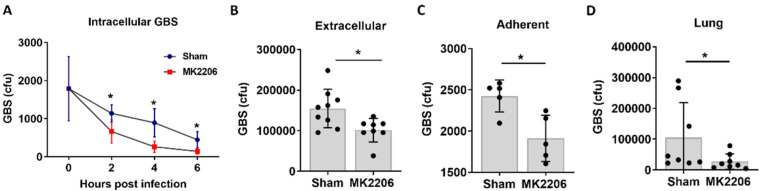
**Akt inhibition promotes Group B *Streptococcus* (GBS) clearance in the lung epithelium.** Alveolar epithelial (A549) cells were treated with the Akt inhibitor (MK-2206) or sham for 24 h and were infected with GBS for 2 h. Intracellular (**A**), extracellular (**B**) and adherent (**C**) GBS colony forming units (cfu) were significantly lower in MK-2206 treated compared to control treated cells at 2 (**A**–**C**), 4 (**A**) and 6 h (**A**) post infection. (**D**). GBS bacterial counts in whole lung homogenates from C57BL/6 mice that were pre-treated with MK2206 by oral gavage and infected with GBS intratracheally. Data are plotted as mean ± S.D (**A**) and median with range (**B**–**D**) and statistical analysis was performed with a Student’s *t*-test (between MK-2206 and the Sham treated cells in each time point) (**A**), and a Mann-Whitney U test (**B**–**D**). n = 5–9 per group.* *p* < 0.05.

To investigate whether the inhibition of Akt facilitates GBS clearance by alveolar epithelial cells, we treated A549 cells with 5 μΜ MK-2206 for 24 h and then infected cells with equal amounts of GBS inoculum for 2 hours. Two hours post infection, the medium containing extracellular GBS was collected, serially diluted and plated. Cells were then replenished with fresh media containing antibiotics to kill any remaining extracellular GBS. At different timepoints (2, 4 and 6 h post infection), cells were collected, and intracellular bacteria were quantified. Adherence of GBS to the epithelial cell surface was also measured at 2 h and calculated as described in the Materials and Methods section. The treatment of A549 cells with MK-2206 resulted in significantly lower counts of extracellular, adherent, and intracellular GBS bacteria compared to control cells (Figure 2A–C), indicating that the inhibition of Akt promotes bacterial clearance by alveolar epithelial cells. 

Next, to test the efficacy of Akt inhibition in GBS clearance in vivo, we utilized a model of GBS pneumonia; 1.5 × 10^6^ GBS were administered intratracheally in C57BL/6 mice previously treated with either MK-2206 (100 mg/kg) via oral gavage or sham. Akt inhibition in lungs upon in vivo MK-2206 treatment was verified with western blot (Supplemental Figure S2). GBS bacterial counts were significantly lower in the lungs of MK-2206 treated mice with GBS infection compared to untreated mice (Figure 2D).

### 2.3. Akt Inhibition Promotes Inflammatory Responses in GBS Infected Epithelial Cells

To investigate the effects of Akt inhibition on inflammatory responses of alveolar epithelial cells during GBS infection, we infected cells as described and measured the induction of inflammatory mediators such as interleukin (IL)-8, IL-6, IL-1β, INFα, Chemokine (C-C motif) ligand (CCL)-2, CCL-5, as well as the production of reactive oxygen species (ROS). Furthermore, the levels of antimicrobial peptides and respiratory ion channels that regulate inflammatory responses and the function of respiratory epithelial cells such as cystic fibrosis transmembrane conductance regulator (CFTR) and surfactant proteins (sp)A–D were also evaluated. 

**Figure 3:**
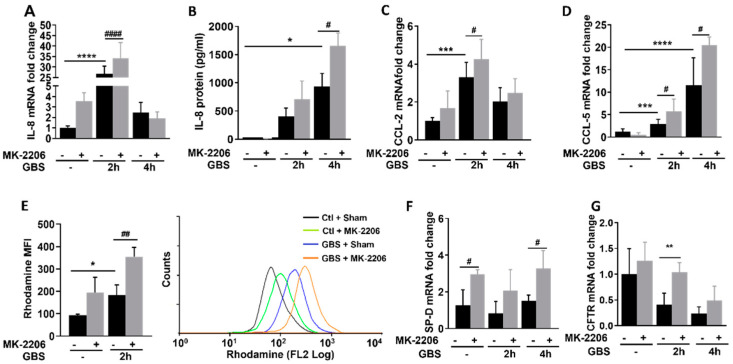
**Akt inhibition induces inflammatory response and production of reactive oxygen species (ROS) in GBS infected alveolar epithelial cells.** Interleukin (IL)-8 mRNA (**A**) and IL-8 protein levels (**B**) and mRNA expression of Chemokine (C-C motif) ligand (CCL)-2 (**C**) and CCL-5 (**D**) upon GBS infection in alveolar epithelial (A549) cells that were pretreated with MK-2206 or sham. (**E**) Reactive oxygen species production in A549 cells treated with MK-2206 or sham, upon GBS infection, measured by rhodamine excitation mean fluorescence intensity (MFI). mRNA levels of surfactant protein D (SP-D) (**F**) and Cystic Fibrosis Transmembrane Receptor (CFTR) (**G**) upon GBS infection in A549 cells that were pretreated with Akt inhibitor MK-2206 or sham. Data are illustrated as bar graphs, plotted as mean ± S.D and statistical analysis was performed using One-Way ANOVA. In rhodamine, flow cytometry assay data are represented in bars, plotted as median with range, and statistical analysis was performed with a Kruskal-Wallis test. n = 6–10 per group. * Comparison among GBS infected cells and control; * *p* < 0.05, ** *p* < 0.01, *** *p* < 0.001, **** *p* < 0.0001. # Comparison among GBS infected cells treated with Akt inhibitor or sham, # *p* < 0.05, ## *p* < 0.01, #### *p* < 0.0001.

IL-8 mRNA and protein levels (Figure 3A,B) as well as CCL-2 and CCL-5 mRNA expression (Figure 3C,D), were significantly induced in GBS infected MK-2206 treated cells compared to GBS infected sham treated cells. Furthermore, using the dihydrorhodamine (DHR) flow cytometry assay, we noticed increased production of ROS in MK-2206 treated compared to sham treated GBS infected epithelial cells (Figure 3E). Furthermore, the expression of the antimicrobial peptide spD and the CFTR channel were elevated in alveolar cells treated with Akt inhibitor (Figure 3F,G). No effect was observed in the expression levels of IL6, IL-1β, INFα and the surfactant proteins A, B and C (Supplemental Figure S3).

### 2.4. Akt Inhibition Induces GBS Clearance in Alveolar Epithelial Cells via Induction of Autophagy

**Figure 4:**
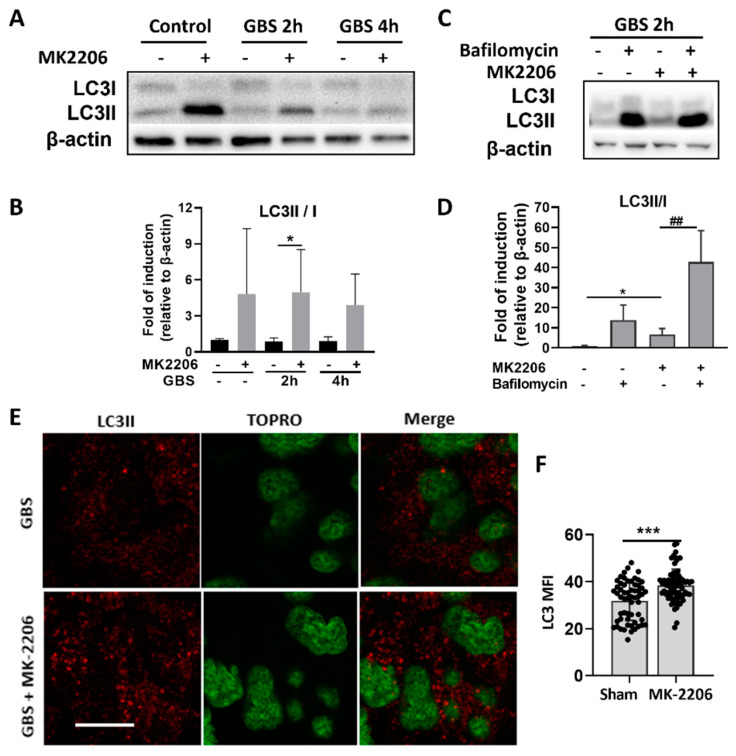
**Akt inhibition induces autophagy and promotes GBS clearance in alveolar epithelial cells**. (**A**,**B**), Representative western blot analysis and densitometry analysis of all replicates of LC3II/I protein levels in control and GBS infected A549 epithelial cells treated with MK-2206 or sham.(**C**,**D**). Representative western blot analysis and densitometry analysis of all replicates of LC3II/I protein levels in control and GBS infected A549 epithelial cells treated with MK-2206 or sham, with or without 200nM bafilomycin, an inhibitor of phagosomal to lysosomal fusion, (**E**,**F**). Representative images of LC3 protein levels and mean fluorescence intensity (MFI) in confocal microscopy of GBS infected A549 cells treated with MK2206 or sham. TOPRO-3 indicates nuclear staining. Bar graph is representative of 10μm. Densitometry analyses are plotted in bar graphs as median +/- interquartile range and statistical analysis was performed using Mann-Whitney test (**B**) or Kruskal-Wallis test with Dunn’s multiple comparison test (**D**), among the groups that are connected with a line, n = 3—6 per group. Data in F are illustrated as bars, plotted as mean ± S.D, and a statistical analysis was performed using an unpaired *t*-test. At least five fields per sample were analyzed per condition with approximately 150 cells per field. MFI was determined by at least 15 regions of interest of stable dimensions per field (n = 60). Results are representative of at least three independent experiments.* *p* < 0.05, ## *p* < 0.01, *** *p* < 0.001.

Autophagy is a well-known process of bacterial clearance regulated by the Akt/mTOR pathway, therefore it was examined as a potential mechanism for the increased bacterial clearance observed upon Akt suppression. As lipidation of LC3 (conversion from LC3I to LC3II) has been established as a useful sign for autophagy, we detected LC3 by immunoblotting and fluorescence microscopy [11]. A significant increase in LC3-II/LC3-I ratio was detected in MK-2206 treated epithelial cells upon GBS infection (Figure 4A,B). LC3-II/LC3-I ratio was substantially increased upon bafilomycin treatment, an inhibitor of phagosomal to lysosomal fusion, indicating activation of autophagy and promotion of lysosomal fusion of GBS cargo, in MK-2206 treated epithelial cells (Figure 4C,D) [25]. Moreover, LC3 aggregates, representing autophagosomes, were elevated in MK-2206 treated epithelial cells as were assessed by immunofluorescence (Figure 4E,F).

**Figure 5:**
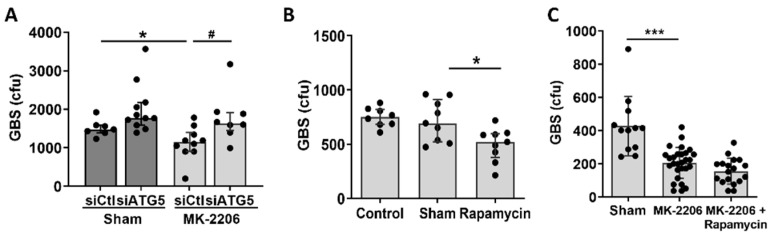
**Induction of autophagy is important for GBS clearance in alveolar epithelial cells.** (**A**). The GBS bacterial load in A549 epithelial cells pre-treated with sham or MK-2206 and which were incubated with control siRNA or siRNA for autophagy-related (ATG)-5, a central regulator of autophagy. (**B**). GBS bacterial load in A549 epithelial cells in which autophagy was induced by rapamycin treatment compared to sham (DMSO diluent) treated cells. (**C**). GBS bacterial load in A549 epithelial cells, treated with MK-2206 or sham, in the presence or absence of rapamycin. Data are illustrated as bars, plotted as median and interquartile range (**A**,**B**) or mean ± S.D (**C**). Statistical analysis was performed using a Student’s *t*-test (**C**) or Mann-Whitney test (**A**,**B**) between the two groups indicated by line. N = 9–24 per group. * or # *p* < 0.05, *** *p* < 0.001.

To further support the role of Akt-mediated autophagy in GBS bacterial clearance in alveolar epithelial cells, we treated A549 cells with silencer (si)RNA targeting Autophagy related (ATG)-5, a central component of the Atg5-Atg12-Atg16L complex, which is required to promote autophagy and LC3 recruitment [15]. Significant knockdown of ATG-5 in cells treated with siATG5 was confirmed with real-time PCR (data not shown). The intracellular bacterial load increased significantly in MK-2206 treated cells in which autophagy was blocked, while no change was observed in sham treated cells (Figure 5A). Furthermore, treatment of A549 cells with rapamycin, which induces autophagy via the suppression of mTOR, resulted in a significant decrease of intracellular GBS bacterial load when compared to cells that received sham treatment (DMSO diluent only) (Figure 5B), indicating that the induction of autophagy enhanced the host defense of alveolar epithelial cells against GBS. Dual suppression of mTOR, with both MK-2206 and rapamycin, did not promote a further decrease in intracellular GBS load compared to MK-2206 treatment alone (Figure 5C).

## 3. Discussion

GBS has the ability to penetrate epithelial barriers and provoke severe invasive disease in neonates and young infants, such as pneumonia, sepsis, and meningitis [26]. In particular, neonates demonstrate “immature” adaptive and innate immune responses, and therefore depend on an enhanced first line of defense and an epithelial barrier defense to combat infections [27]. Today, little is known about the host defenses employed by non-professional phagocytes, such as the alveolar epithelial cells, which, in the case of GBS infection, serve as the site of entry to the bloodstream. Furthermore, it is unknown whether we can enhance the bactericidal capacity of lung epithelial cells against GBS. 

In the present study, we sought to investigate whether we can enhance the inflammatory responses and bacterial killing capacity of alveolar epithelial cells in order to hamper GBS blood invasion and improve survival from neonatal GBS sepsis. Specifically, we focused on the role of Akt kinase, since previous studies found that Akt deficient phagocytic cells have an enhanced bactericidal capacity mediated by the induction of autophagy, which is regulated by the suppression of the Akt/mTOR pathway [21,24]. To investigate the effects of Akt inhibition on alveolar epithelial cells, we treated the alveolar epithelial cell line A549 with the allosteric specific Akt inhibitor MK-2206, which is known to inhibit the phosphorylation and activation of Akt kinase at Τ308 and S473 [28]. Treatment of A549 cells with the appropriate concentration of MK-2206 for 24 h was sufficient to inhibit Akt kinase without compromising cell proliferation. 

Based on our results, GBS activates Akt in alveolar epithelial cells and Akt inhibition is important for clearance of extracellular, adherent and intracellular GBS. Indeed, the PI3K/Akt pathway is activated when various pathogens bind to cell surface receptors such as TLRs and several pathogens utilize the Akt pathway to promote cell invasion and survival [29,30,31]. GBS has been shown to bind to TLR2 and activate PI3K/Akt signaling [28]. The importance of Akt for survival of other pathogens has also been shown in studies in macrophages or endothelial cells; inhibitors of Akt have been shown to prevent intracellular growth of various intracellular bacteria such as *Salmonella typhimurium*, *Mycobacterium tuberculosis* and *Leismania* spp [20,32]. However, this is the first report to identify that Akt inhibition facilitates the clearance of GBS and has the ability to enhance the innate immune responses of alveolar epithelial cells. 

Indeed, the effect of Akt inhibition on the GBS bacterial load was evident in both extracellular and intracellular bacteria. We found that the airway epithelial cells responded by increasing the production of mediators such as cytokines (IL-8), chemokines (CCL-2,5), oxidative stress, antimicrobial peptides (SP-D) and ion channels (CFTR) upon Akt inhibition and GBS exposure. These mediators are important local innate immune effectors and contribute to killing of microorganisms by non-phagocytic cells [33,34,35,36]. For example, SP-D is a lung epithelial collectin that functions as a soluble pattern recognition receptor in the airspaces and opsonize or aggregate microbes [37]. Additionally, IL-8 and the chemokines CCL-2,5 are important for recruiting neutrophils, monocytes, and T lymphocytes at the site of infection [33,34,35]. The extracellular effect of Akt in GBS is also essential in preventing invasive disease, as it reduces the initial inoculum, the adherence of GBS to the epithelium, and may hamper GBS penetration to the bloodstream by paracellular routes [38].

Apart from its effect on the extracellular bacteria, we also observed a significant reduction in the intracellular GBS load in epithelial cells upon Akt inhibition and we showed that activation of autophagy was important in this process. Recently, the role of autophagy as a cell defense towards invading bacteria has gathered increasing interest [39]. Xenophagy is an evolutionarily conserved mechanism where components of the autophagic machinery are recruited to facilitate intracellular pathogen elimination and degradation via fusion with the lysosome [11]. Previous studies showed that the induction of autophagy can protect alveolar epithelial cells from intracellular respiratory pathogens, such as *Mycobacterium tuberculosis* and Group A *Streptococcus*, as well as extracellular pathogens such as *Pseudomonas aeruginosa* and *Streptococcus pneumoniae* [12,40,41]. Indeed, many pathogens have evolved to avoid their destruction by inhibiting the autophagic response or subverting the autophagic pathway for prolonged intracellular survival within phagosomes [9,42,43]. 

In the present study, we examined whether autophagy is the mechanism that is responsible for the enhanced bactericidal capacity observed in A549 cells with the inhibition of Akt activity. The inhibition of Akt activity using MK-2206 resulted in the induction of the autophagic mechanism, as was shown by the increased LC3II/I ratio and increased autophagic flux in MK2206 treated cells. To further support the induction of autophagy in A549 cells subjected to Akt inhibition, we identified LC3 protein levels with confocal microscopy and confirmed the significantly elevated LC3 punctation in MK2206 treated cells. The increased production of ROS observed also correlates with autophagy induction, since it has been shown that ROS activate autophagy to protect cells from nutrient starvation, cell death, and invading pathogens [44]. In accordance with our findings, Akt inhibition is known to induce autophagy, via mTOR, a downstream target of the PI3K/Akt pathway, that is known to inhibit autophagy [45]. Activated Akt phosphorylates and thereby inhibits the TSC1/2 complex, which dissociates from the lysosome, allowing Rheb activation, which is a potent mTORC1 activator [46,47,48]. Thus, when Akt activation is inhibited with MK2206, the TSC1/2 complex is not inhibited and does not allow Rheb and consequently mTORC1 activation, rendering it unable to inhibit autophagy [49]. Studies have shown that several pathogens, such as *Streptococcus pneumoniae*, exploit this mechanism naturally [50]. Pathogen-induced host membrane damage, either directly from pathogens or indirectly from their secreted toxic virulence factors, cause an acute intracellular amino acid starvation [49,51]. This amino acid starvation is perceived by AMP-activated protein kinase (AMPK), which causes the downregulation of mTOR activity, resulting in the induction of autophagy [52]. Other studies have shown that the autophagic machinery could be exploited to effectively eliminate pathogenic Group A *Streptococcus* within non-phagocytic cells [12]. Additionally, other pathogens such as *Salmonella typhimurium* activate the Akt pathway to achieve survival in phagocytic cells [30]. The enhanced bacterial clearance mediated through autophagy was also confirmed with the suppression of autophagy by the silencing of ATG5 and the use of rapamycin, which targets mTORC1 and induces autophagy. The blockage of autophagy abrogated the protective effect of Akt inhibition in alveolar epithelial cells while rapamycin pre-treatment enhanced the bactericidal capacity of alveolar epithelial cells. Therefore, autophagy serves as an antibacterial mechanism for alveolar epithelial cells against GBS infection. The survival benefit towards GBS infection in Akt deficiency was also confirmed in vivo, as we observed a significantly lower bacterial load in the lungs of mice with GBS pneumonia in which the chemical inhibition of Akt had been performed, indicating that Akt inhibition in the epithelium could enhance the lung host defense against GBS. 

Collectively, our data demonstrate that inhibition of Akt kinase in GBS infected alveolar epithelial cells results in augmented inflammatory responses, the induction of autophagy and enhanced GBS clearance. The enhanced autophagy-mediated killing in non-phagocytic cells may hamper GBS invasion from the alveoli to the bloodstream, leading to the prevention of severe GBS disease in susceptible hosts. 

## 4. Materials and Methods

### 4.1. GBS Strain and Culture

For experimental infection, *Streptococcus agalactiae* (GBS) serotype III, which belongs to the hypervirulent clone ST-17, isolated from a neonate with sepsis and meningitis, was kindly provided by Prof. Petinaki [53]. Bacteria were grown in Todd Hewitt (TH) broth (Oxoid, Basingstoke, UK) supplemented with 0.5% yeast extract (Sigma-Aldrich, St. Louis, MO, USA) and *Streptococcus* selective medium (5 ug/mL Colistin-Sulphate, 0.5 ug/mL Oxalinic acid, Streptococcus Selective Supplement, Oxoid, Basingstoke, UK) and maintained in stock cultures in 30% glycerol at −80 °C. Before the experiment, bacteria from frozen stocks were inoculated in TH agar plates and incubated at 37 °C overnight. Working cultures were made by growing single colonies in 50 mL of TH broth to mid-log exponential phase (optical density of 0.5 to 0.7 at 600 nm). The bacteria were washed twice in phosphate-buffered saline (PBS), pH 7.4, and adjusted photometrically to the desired concentration. The verification of experimental inoculums was performed in each experiment via the determination of colony form units (cfu).

### 4.2. GBS Survival Assay in Alveolar Epithelial Cells

The A549 cell line (ATCC: CCL-185) was used in this study as a model of type II pulmonary epithelial cells [54]. Cells were cultured in Dulbecco’s Modified Eagle Medium (DMEM), low glucose (1 g/L) (Thermo Fisher Scientific, Waltham, MA, USA) supplemented with 10% fetal bovine serum (FBS), 5 μg/ml Penicillin G (Sigma-Aldrich, St. Louis, MO, USA) and 100 μg/ml Gentamicin (Sigma-Aldrich, St. Louis, MO, USA) at 37 °C in 5% CO_2_. A549 cells were seeded at a final density of 5 × 10^5^ cells/well in 24 well plates and were pre-treated with the selective Akt 1/2 inhibitor MK-2206 2HCl (SelleckChem, Berlin, Germany) at different concentrations (2.5, 5, 10 μΜ) and its solvent dimethyl sulphoxide (DMSO, Sigma-Aldrich, St. Louis, MO, USA) for 24 h at 37 °C in 5% CO_2_. In specified experiments, A549 cells were pretreated with 5 μΜ rapamycin (Cayman Chemical, Michigan, MI, USA) or 200 nM Bafilomycin (Sigma-Aldrich, St. Louis, MO, USA) for 2 h prior to GBS infection. GBS was grown to the mid-log phase in TH broth and was diluted to the appropriate concentration in sterile normal saline. Cells were washed three times prior to GBS infection. GBS was centrifuged at 6000 rpm, resuspended in DMEM plus 10% FBS and was administered in A549 cells at a multiplicity of infection of 10 bacteria/cell (MOI 10:1) for 2 h at 3 °C. 

Bacteria were allowed to be internalized by the cells for 2 h at 37 °C. After this time (time zero of the assay), the extracellular medium containing extracellular GBS was serially diluted and plated. Cells were then washed three times with PBS, and the medium was replaced with DMEM containing 10% FBS, penicillin G (5 μg/mL) and gentamycin (100 μg/mL) to kill extracellular GBS. At specific timepoints, cells were detached from the plate with 0.25% Trypsin-EDTA (Gibco, MA, USA) and lysed with 0.025% Triton X to release intracellular bacteria (Sigma-Aldrich, St. Louis, MO, USA). Adherence of GBS to the epithelial cell surface was also measured at 2 h post infection by collecting and lysing cells before the addition of antibiotics in the cell medium. The numbers of adherent bacteria were then calculated as follows: total bacterial load of lysed cells minus the intracellular bacterial load. The lysates were then serially diluted in water and plated on TH broth dishes. The plates were placed at 37^o^C overnight and the quantification of the intracellular bacterial load in each inoculum was estimated through colony-forming unit (cfu) counting. 

### 4.3. Murine Model of GBS Pneumonia

C57BL/6 mice were kept in the animal facility of the School of Medicine, University of Crete, in a temperature-controlled room and twelve-hour light/dark cycle, with free access to standard laboratory chow and water. For in vivo experiments we used adult mice (8–10 weeks old). For Akt inhibition, 100 mg/kg MK-2206 dissolved in 15% Captisol (SelleckChem, Berlin, Germany) was used. MK-2206 was administered by oral gavage 3 h prior to infection, while the control group received 15% Captisol in normal saline. All mice were then infected with 1.5 × 10^6^ GBS intratracheally for 10–12 h. 

### 4.4. In Vivo Murine Sample Collection, Analysis, and Survival Experiments

At specific time points following GBS administration, mice were anaesthetized with i.p ketamine (100 mg/kg) and xylazine (8 mg/kg), and lung tissues were harvested for analysis. Prior to lung tissue collection, mice were perfused with ice cold PBS via the left ventricle and bronchoalveolar lavage was performed to remove alveolar macrophages. Whole lungs were then briefly immersed in 70% ethanol and sterile water, and then disrupted with a mortar and pestle. The homogenized tissues were suspended in 500 μL of Triton X 0.05% in sterile PBS solution for further cell lysis. Suspensions were serially diluted, plated on TH agar plates and incubated at 37 °C for 24h. Results are reported in cfu per milliliter/per tissue.

### 4.5. Cell Proliferation Assay

A549 cells were seeded at a density of 1 × 10^4^ cells/well in a 96-well plate and treated with 5 and 10 μM MK-2206 and DMSO for 48 h. At each timepoint, 11 μL of MTT reagent (5 mg/mL) per 100 μL of cell culture medium was added, and 4 h later the medium was removed and 100 μL isopropanol/HCl was also added into the wells. Cells were incubated for 5 min in a shaker incubator and then OD at 550m was measured in a microplate reader iMark (Bio Rad, Hercules, CA, USA). The cell proliferation rate was calculated by dividing the OD of the MK-2206 treated cells by the OD of the control cells. 

### 4.6. RNA Isolation and Real-Time PCR 

Total RNA was extracted using TRIzol reagent (Life Technologies, Carlsbad, CA, USA) and quantified by spectrometry at 260 and 280nm. One microgram of total DNA-digested RNA was used for cDNA synthesis (TAKARA, Primescript RT Reagent kit, Tokyo, Japan). Amplification was performed using KAPA SyBr® Fast Universal qPCR kit (Kapa Biosystems, Cape Town, South Africa). The SYBR Green method was followed in the PCR reaction. Denaturation was carried out at 95 °C for 10 s, annealing and extension at 60 °C for 30 s for 40 cycles in a StepOnePlus™ Real-Time PCR System (Applied Biosystems, Foster City, CA, USA). Data analysis was accomplished using the ΔΔCT method and GAPDH was used as the housekeeping gene. The primer sequences used in this study are provided in Supplemental Table S1.

### 4.7. Enzyme-Linked Immunoabsorbent Assay (ELISA) 

Cell culture supernatants were collected after infection for cytokine quantification. Cytokine concentration for IL-8 was determined at 12 and 24 h post GBS infection using the Human IL8 Elisa Max™ Delux Set (BioLegend, SanDiego, CA, USA) following the manufacturer’s instructions.

### 4.8. Western Blot

For protein extraction, cell lysates were harvested with RIPA lysis buffer (10 mM Tris-HCl pH 8.0, 10 mM EDTA, 140 mM NaCl, 1% Triton X-100, 1% Na Deoxycholate, 0.1% SDS), supplemented with Protease Inhibitors (Protease Inhibitor complete tablets, Roché, Basel, Switzerland) and phosphatase inhibitors (Sigma-Aldrich, St. Louis, MO, USA). Protein concentration was determined using the BCA Protein Assay (Sigma-Aldrich, St. Louis, MO, USA). Protein lysates were resuspended in SDS-containing loading dye, were separated on 13.3% or 10% polyacrylamide gel, and then transferred to nitrocellulose membrane. Briefly, after blocking with 5% Bovine Serum Albumin (BSA) containing 0.1% Tween 20 for an hour at room temperature, the membranes were incubated overnight at 4 °C with primary antibodies (1:1000), washed with PBST and then incubated with peroxidase-conjugated secondary antibodies (1:5000) for 1 h at room temperature. The antibodies used in this study were the following: p-Akt (Cell Signaling, Danvers, MA, USA, #4060), Akt (Cell Signaling, #9272), LC3B (Cell Signaling, #3868), p-S6 (Cell Signaling, #2211), S6 (Cell Signaling, #2217), p-4EBP1 (Cell Signaling, #2855), 4EBP1 (Cell Signaling, #9644), and β-actin (Cell Signaling, #3700). The visualization of membranes was performed using Lumi-Light ECL substrate (Thermo Fisher Scientific, Waltham, MA, USA). Membranes were exposed to trans-UV light in a ChemiDoc XRS+ (BioRad Laboratories, Inc, Hercules, CA, USA), and signals were digitalized and analyzed by densitometry with the relevant software (Image Lab Software, v.6.1, Biorad, CA, USA). The band intensities of phosphorylated proteins were normalized with total protein intensities as well as with the loading control beta-actin. 

### 4.9. Gene Silencing 

For gene silencing experiments, A549 alveolar epithelial cells were seeded in 96-well tissue culture plates in a volume of 0.1 mL complete medium (DMEM; Life Technologies) supplemented with 10% (*v*/*v*) FBS, 10 mM L-glutamine, 5 μg/mL Penicillin G, and 100 μg/mL Gentamicin. Cells were incubated at 37 °C for 4 h, and the medium was replaced with serum-free, antibiotic-free DMEM prior to transfection. Lipofectamine served as transfection reagent (RNAiMAX; Thermofisher Scientific, Waltham, MA, USA) and cells were transfected with either 50 nM small interfering RNA (siRNA) for Atg5 (Silencer Select siRNAs, Thermofisher Scientific, Waltham, MA, USA) or with negative-control siRNA. Transfection efficiency and biological effect were assessed 72 h post-transfection.

### 4.10. Measurement of Reactive Oxygen Species

Reactive oxygen species (ROS) production in A549 cells was determined using the fluorogenic dye Dihydrorhodamine 123 (DHR 123) in flow cytometry. After cell uptake, DHR 123 is oxidized by ROS into a fluorescent compound (Rhodamine 123) that can be detected by a flow cytometer with a maximum excitation and emission spectra of 500 and 536 nm, respectively. A549 cells were seeded at a density of 6.5 × 10^5^ cells/well in a 24-well plate and treated with 5μM MK-2206 and DMSO for 24 hours. Cells were stimulated with GBS at a MOI of 10:1 and the cell permeable dye D-123 was added at a concentration of 0.2ug/mL (Millipore, St. Louis, MO, USA) in antibiotic-free cell medium. After 2 h of phagocytosis, antibiotics were added to the cell medium and at the desired time points after GBS infection, the reaction was terminated by adding cold PBS in each well, cells were washed and detached with a scrapper, centrifuged for 5 min at 500g at 4 ^o^C, and fixed with 2% PFA in PBS. The mean fluorescence intensity (MFI) of DHR was assessed by flow cytometry in a FACS Calibur (BD Biosciences, San Jose, CA, USA) and analyzed with the use of Summit v4.3 Software (Dako, Agilent technologies, CA, USA).

### 4.11. Immunofluorescence Staining

A549 cells were seeded at a density of 2 × 10^5^ cells/well in glass coverslips into a 24-well plate and treated with 5μM MK-2206 and DMSO for 24 h. GBS infection followed for 2 h and then cells were washed and fixed with methanol for 10 min. Cells were incubated in a blocking buffer (5% FBS, 1% BSA, 0.3% Triton-Χ 100 in PBS) for 30 min and stained with anti-LC3II rabbit antibody (cat#L7543, Sigma-Aldrich, St. Louis, MO, USA) diluted at 1:800 for 1 h. Cells were then incubated with an anti-rabbit IgG conjugated with CF-488A (cat#SAB4600042, Sigma-Aldrich, St. Louis, MO, USA) diluted at 1:600 for 40 min and a DNA stain was carried out with TO-PRO-3 Iodide (Invitrogen, Massachusetts, USA) diluted at 1:1000 for 10 min under darkness. Immunofluorescence was examined using fluorescence microscopy (Leica SP8) and images were analyzed in Las X software.

### 4.12. Statistical Analysis 

All numeric data were evaluated for normality using the Kolmogorov–Smirnov test. Comparison among groups was performed using *t*-test and one-way-ANOVA for parametric data, or Mann – Whitney and the Kruskal — Wallis test with Dunn’s multiple comparison post-test for non-parametric data. Data were depicted in bars, or dot plots and plotted as mean ± SD or as median and interquartile range. The GraphPad InStat Software (GraphPad v8.0, San Diego, CA, USA) was used for analysis. *p* value < 0.05 was considered statistically significant. Results are representative of at least three independent experiments.

## Data Availability

All data associated with this study are available in the main text or the Supplementary Materials. Data that are not shown are available upon request.

## Supplementary Materials

The following supporting information can be downloaded at: https://www.mdpi.com/article/10.3390/pathogens11101134/s1, Table S1. Primer sequences used in this study. Figure S1: Effect of Akt inhibition on alveolar epithelial cell proliferation, Figure S2. Suppression of p-Akt in vivo by MK-2206 treatment, Figure S3. Expression of IL6, IL-1β, INFα and surfactants by A549 epithelial cells upon GBS infection and Ak inhibition.
