# Supplementary material for: Akt Inhibition Promotes Autophagy and Clearance of Group B Streptococcus from the Alveolar Epithelium
Source: Pathogens. 2022 Sep 30;11(10):1134. doi: 10.3390/pathogens11101134 (PMC9611837; doi:10.3390/pathogens11101134)
Supplement: Supplementary file 1 [file pathogens-11-01134-s001.zip › pathogens-1874906-supplementary.pdf]

Supplementary Material

Supplementary Table S1. Primer sequences used in this study.

Gene		Sequence (5′-3′)
IL8	Forward	TGTGAAGGTGCAGTTTTGCC
	Reverse	CACCCAGTTTTCCTTGGGGT
CCL2	Forward	GCTCAGCCAGATGCAATCAA
	Reverse	TGAACCCACTTCTGCTTGGG
CCL5	Forward	CCAGCAGTCGTCCACAGGT
	Reverse	ACACTTGGCGGTTCTTTGGG
ATG5	Forward	TGACGTTGGTAACTGACAAAGT
	Reverse	AATGCCATTTCAG-TGGTGTG
CFTR	Forward	AAAAGGCCAGCGTTGTCTCC
	Reverse	GCTCTCTATCCCATTCTCTTTCCA
SP-A	Forward	CATGGGTCCACCTGGAGAAA
	Reverse	AAAGTCGTGGAGTGTGGCTT
SP-B	Forward	AAGCCATGATTCCCAAGGGTG
	Reverse	CATTCTCCTGTCGGCGACCT
SP-C	Forward	GAGCCAGAAACACACGGAGA
	Reverse	GTGAGAGCCTCAAGACTGGG
SP-D	Forward	ATTGAAGGGGGACAAAGGCAT
	Reverse	ATTTGGGAAGAGCTCAACTTTCTTA
INFα	Forward	GGAGGAGAGGGTGGGAGAAA
	Reverse	GACAACCTCCCAGGCACAAG
IL1-β	Forward	CGGACCCCAAAAGATGAAGGGCTG
	Reverse	GCTCTTGTTGATGTGCTGCTGCGAG
IL6	Forward	GTCAGGGGTGGTTATTGCAT
	Reverse	AGTGAGGAACAAGCCAGAGC
GAPDH	Forward	GGAAGGTGAAGGTCGGAGTCA
	Reverse	GTCATT-GATGGCAACAATATCCACT

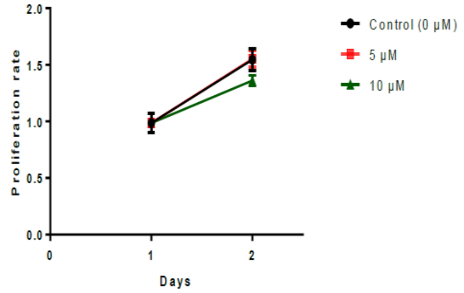

Supplementary Figure S1. **Effect of Akt inhibition on alveolar epithelial cell proliferation**. Cell proliferation was determined using MTT assay in A549 treated with MK2206 (5, 10 μM). Higher concentration of MK2206 (10μM) negatively impacts cell proliferation.

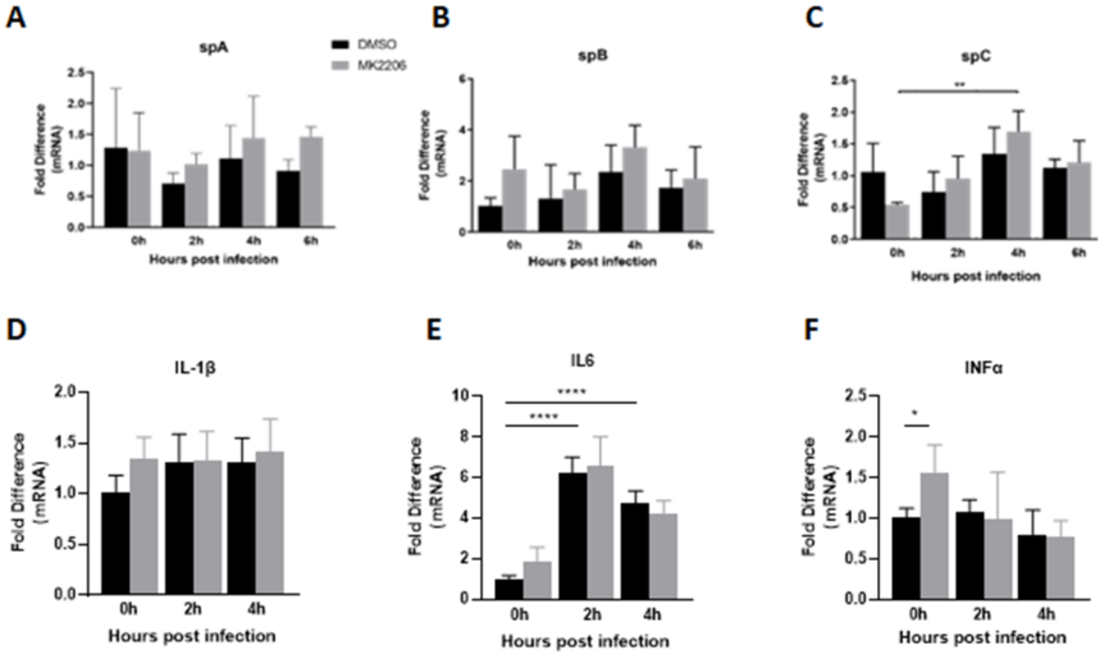

Supplemental Figure S2. Suppression of p-Akt *in vivo* by MK-2206 treatment. A, Representative western blot analysis of Akt phosphorylated levels in mice with GBS pneumonia that were treated with MK-2206 compared to sham. Data are illustrated as bars, and plotted as median with range and statistical analysis was performed with Mann-Whitney (n=4). *p < 0.05.

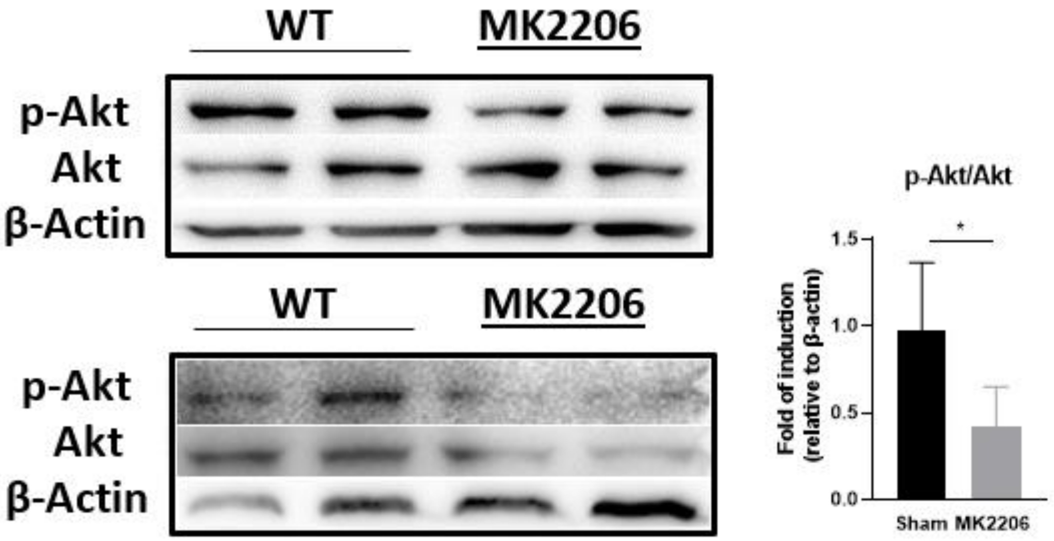

Supplementary Figure S3. Expression of surfactants by A549 epithelial cells upon GBS infection and Ak inhibition. A549 cells were treated with MK2206 or sham for 24 hours and infected with GBS for 2 hours. Expression (mRNA) of the surfactant proteins sp-A, sp-B and sp-C (A, B, C), and IL-1β, IL6 and INFα (D, E, F) upon pre-treatment with MK2206 and GBS infection was observed. Data are illustrated as bars, plotted as mean ± S.D and statistical analysis performed using One-Way ANOVA. *p < 0.05, **p < 0.01, ****p<0.0001.
